# Delayed onsets are not necessary for generating distractor quitting thresholds effects in visual search

**DOI:** 10.3758/s13414-023-02734-0

**Published:** 2023-07-06

**Authors:** Rebecca K. Lawrence, Karlien H. W. Paas, Brett A. Cochrane, Jay Pratt

**Affiliations:** 1 School of Applied Psychology, Griffith University, Level 7 Ian O’Connor Building (G40), Parklands Drive, Southport, Qld 4222 Australia; 2 University of Aberdeen, Aberdeen, UK; 3 The University of Toronto, Toronto, Canada

**Keywords:** Attentional capture, Visual search

## Abstract

Salient distractors lower quitting thresholds in visual search. That is, when searching for the presence of a target among filler items, a large heterogeneously coloured distractor presented at a delayed onset produces quick target-absent judgements and increased target-present errors. The aim of the current study was to explore if the timing of the salient distractor modulates this Quitting Threshold Effect (QTE). In Experiment 1, participants completed a target detection search task in the presence or absence of a salient singleton distractor that either appeared simultaneously with other search items or appeared at a delayed onset (i.e., 100 ms or 250 ms after other array items appeared). In Experiment 2, a similar method was used, except that the salient singleton distractor appeared simultaneously, 100 ms before, or 100 ms after the other array items. Across both experiments, we observed robust distractor QTEs. Regardless of their onset, salient distractors decreased target-absent search speeds and increased target-present error rates. In all, the present findings suggest that delayed onsets are not required for lowered quitting thresholds in visual search.

When a target is present is a visual search task, it has long been known that highly salient distractors can capture attention and interfere with the search process (e.g., Theeuwes, 1992). But what is the impact of such distractors on visual searches in which the target is absent? This question was asked by Moher (2020) who had participants determine if a vertical blue rectangle (the target) was present or absent in a search array containing diagonally oriented blue rectangles of the same size. The target was present on 50% of trials, and absent on 50% of the trials. To explore the effect of highly distracting information on search, on 50% of all trials, Moher replaced one of the diagonal blue rectangles with a much larger red rectangle that was also tilted (the distractor). Further, to maximise the salience of this distractor, the large red rectangle also had a delayed onset, appearing 100 ms after all other items in the search array. As expected, the distractor slowed search speeds and increased errors in target-present trials. Critically, Moher found the distractor speeded responses when the target was absent.

To account for these findings, Moher (2020) proposed that the salient distractor influenced participant quitting thresholds. A quitting threshold reflects the amount of evidence used by the observer to decide whether the target is absent from the visual search array (Wolfe, 2021; Wolfe & Van Wert, 2010). Several factors have been found to influence quitting thresholds such as the average amount of time a participant spends searching for a target (Becker et al., 2022), information held in working memory (Wu & Pan, 2022), and perhaps most well-known, target prevalence, where low prevalence results in lowered quitting thresholds (e.g., Wolfe & Van Wert, 2010). Furthermore, among other factors, Peltier and Becker (2017) found that higher levels of introversion were associated with increased quitting thresholds for low prevalence visual search tasks.

In Moher (2020), given that the red rectangle both increased errors for target-present trials and sped search for target-absent trials, it appears likely that the distractor lowered the amount of evidence observers considered before ending each search, corresponding to a lower quitting threshold. One explanation for this effect is that during search, the participants may have inspected and reinspected the salient distractor multiple times during a trial. Given the salience of the distractor, it may have been memorable, and thus, reinspection might have caused swift search termination and occasional target misses. That is, one might realise that they had processed the salient distractor more than once, therefore concluding that the entire array had been inspected, and, therefore, that the target was absent (Lawrence & Pratt, 2022; Moher, 2020; Moran et al., 2013; Wolfe, 2021).

Extending on the work of Moher (2020), Lawrence and Pratt (2022) recently explored how the overall salience of the distractor might influence this Quitting Threshold Effect (QTE). Here, a similar method to Moher (2020) was used, except that the salience of the distractor was manipulated across experiments by varying its size. Specifically, in Experiment 1, participants completed the search task in the presence or absence of a large, delayed-onset red rectangle distractor. However, in Experiment 2, the search task was completed in the presence or absence of a smaller, delayed-onset red rectangle distractor. Critically, the salience of the distractor mattered. When a larger distractor rectangle was present, it lowered quitting thresholds during visual search. However, when the smaller rectangle was present, the quitting threshold was unaffected. Instead, the distractor slowed down both-target-present and target-absent visual searches. These results suggest that the singleton distractor must be highly salient for the QTE to emerge.

Another factor thought to maximise salience that was utilised in both Moher (2020) and Lawrence and Pratt (2022) was delaying the onset of the salient distractor. As noted, the salient distractor appeared 100 ms after the appearance of all the other items in the search array. Indeed, Moher (2020) specifically noted that “This was done to make the distractor as salient as possible” (p. 33), and his choice was based on the well-known finding that abrupt onsets can capture attention (Jonides & Yantis, 1988; Remington et al., 1992; Yantis & Jonides, 1984). This choice leads to an interesting possibility; if quitting thresholds are modulated by distractor salience, and distractor onset modulates distractor salience, then changes in onset delays should influence the QTE.

As such, the current study evaluated whether the timing of the singleton distractor modulates the QTE. To do so, we used a visual search task conceptually identical to that used in both Moher (2020) and Lawrence and Pratt (2022), except that the distractor onset varied. Following the results of Lawrence and Pratt, as distractor onsets differ more from the onset of the other display items, salience should increase, which should increase the QTE.

## Experiment 1

Experiment 1 tested the effect of three different distractor onsets on the QTE. A similar method to that used in Moher (2020) was adopted, except that the distractor could appear at the same time as the other array items (0 ms condition) or was delayed by either 100 ms or 250 ms after the other array items. Given that Moher used a delayed onset to maximise distractor salience, we expected to see larger QTEs for the 100 ms and 250 ms delayed onset conditions compared with the 0 ms onset condition.

### Method

#### Participants

One-hundred and nine participants completed an online demographic survey and visual search task (*M* = 22.73 years, *SD* = 6.36 years).^1^ Eighty-seven were female, 20 were male, one was other, and one did not report this information. Ninety-four were right-handed, 12 were left-handed, two were ambidextrous, and one did not report handedness. One-hundred-and-six reported normal or corrected-to-normal vision, two reported having vision problems, and one chose not to respond. All participants provided informed consent before participation and were compensated with course credit for an undergraduate psychology course.

#### Materials and procedure

**Fig. 1:**
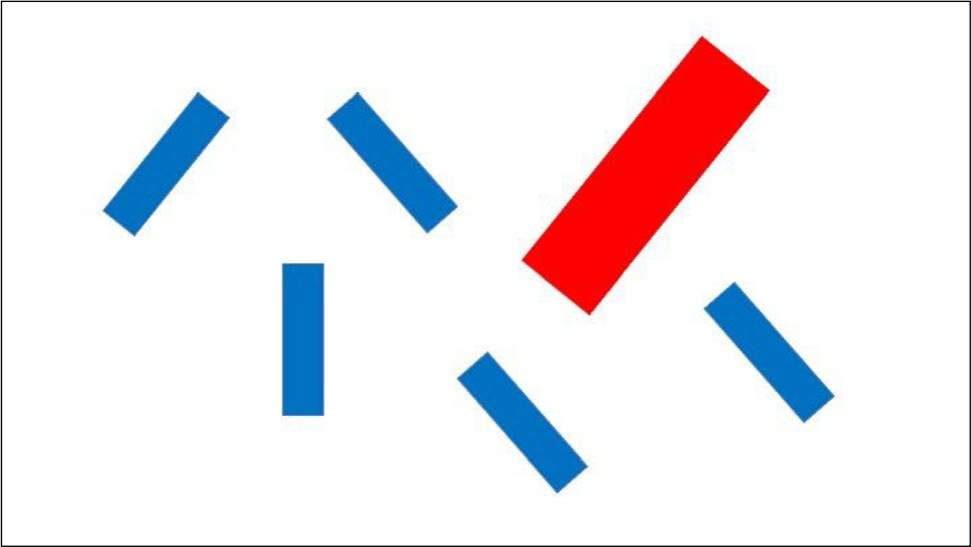
An example of a typical search trial for Experiment 1 and 2. In particular, Fig. 1 depicts a trial when both the target (vertical blue rectangle) and salient singleton distractor (red tilted rectangle) are present. (Colour figure online)

The study was conducted online. After providing informed consent, participants first completed a demographic survey which was created using Lime Survey (limesurvey.org). Next, they completed a visual search task programmed on PsychoPy and presented on Pavlovia (Peirce et al., 2019). A typical search array is depicted in Fig. 1. At the beginning of each trial, participants saw a white screen for 1,500 ms. Next, an array of six rectangles appeared on the computer screen. The location of each rectangle was randomly determined on each trial by drawing an invisible 500- by 500-pixel grid centred on the middle of the computer screen, with grid lines spaced 50 pixels apart (the rectangles could appear anywhere that two grid lines intersected).

For the target-absent trials (50% of trials), all six rectangles presented were randomly tilted by 30 degrees or 330 degrees (filler rectangles). However, for the target-present trials (50% of trials), one of the rectangles was vertically oriented (i.e., 0 degrees and acted as a target). Furthermore, on 50% of all trials, the target and filler rectangles were 40 by 8 pixels in size and coloured blue. However, on the other 50% of trials, one of the blue filler rectangles was replaced by a large red tilted rectangle that was 80 by 16 pixels. Critically, the onset of this salient distractor rectangle was also varied throughout the experiment. It appeared either at the same time (0ms), 100 ms after, or 250 ms after the onset of the search array. On each trial, the participants determined if the target rectangle was present or absent by pressing “z” or “m” on their keyboard, respectively. All items remained on the screen until a response was made. During both the practice and experimental trials, a small message reminding participants of the task instructions was presented at the bottom of the screen.

The experimental session lasted approximately 40 minutes and consisted of 12 practice trials with corrective feedback and 300 experimental trials. The experimental trials were broken into three blocks of 100 trials, and distractor onset was held constant within a block (i.e., one block for the 0-ms onset condition, one block for the 100-ms onset condition, and one block for the 250-ms onset condition). During each block, each combination of target presence and distractor presence occurred 25 times each with the order of the trial type randomised. Thus, throughout the experiment, there were 12 possible trial types—that is, target (present/absent), distractor (present/absent), distractor onset (0 ms/100 ms/250 ms). The order of the three distractor onset blocks was randomly determined for each participant and the experimental blocking was used to statistically analyse the effect of onset (i.e., the experiment block was included as a factor in analyses to reflect the effect of onset). Rest breaks were offered after each experimental block and at the end of the experimental session, participants were debriefed.

### Results

#### Data cleaning

Before conducting the main statistical analyses, error rate and correct response time (RT) data was screened. Similar to the process used by Moher (2020), participants were removed from further analyses if their accuracy was lower than 60% across the experiment (*N* = 3) or if their accuracy for any one experimental condition was lower than 10%, which would indicate systematically incorrect responses (*N* = 1). Next, the distributions and extreme scores for error rates were examined at the group level. Here, outliers were determined as participants whose mean accuracy in any condition of the experiment exceeded ±3.29 standard deviations from the group mean for that condition (i.e., the most extreme 0.1% of participants for that condition) and were removed from the data set (*N* =19; Tabachnick & Fidell, 2013). After this, correct RTs were examined. At the individual level, we excluded trials where participants responded faster than 200 ms or slower than 10 seconds (Moher, 2020). On average, this led to the removal of 0.28% (*SD* = 1.04%) of correct trials per participant. Finally, at the group level, we checked for univariate outliers for the mean correct RT data, with participant RTs exceeding +/- 3.29 SDs of the mean RT in any condition being excluded (*N* =2). These exclusions left a final sample of 84 participants.

Across both Experiments, we report traditional frequentist statistics as well as supplementary Bayes factors (for both ANOVA and *t* tests). The frequentist statistics were conducted using SPSS, and for repeated-measures ANOVA, when assumption of sphericity was violated, Greenhouse–Geisser values were reported. The Bayesian statistics were conducted using JASP Version 0.17.1. (JASP Team, 2023; Morey & Rouder, 2015; Rouder et al., 2009, 2012). For repeated-measures Bayesian ANOVAs, priors were uniform such that were set at 0.20 for each model. Bayes factors were pooled across matched models and reported for each effect of interest (i.e., distractor, onset, distractor * onset). For Bayesian paired-samples *t* tests, the alternative hypothesis was nondirectional, and the default prior was used.

#### Target-present trials

**Fig. 2:**
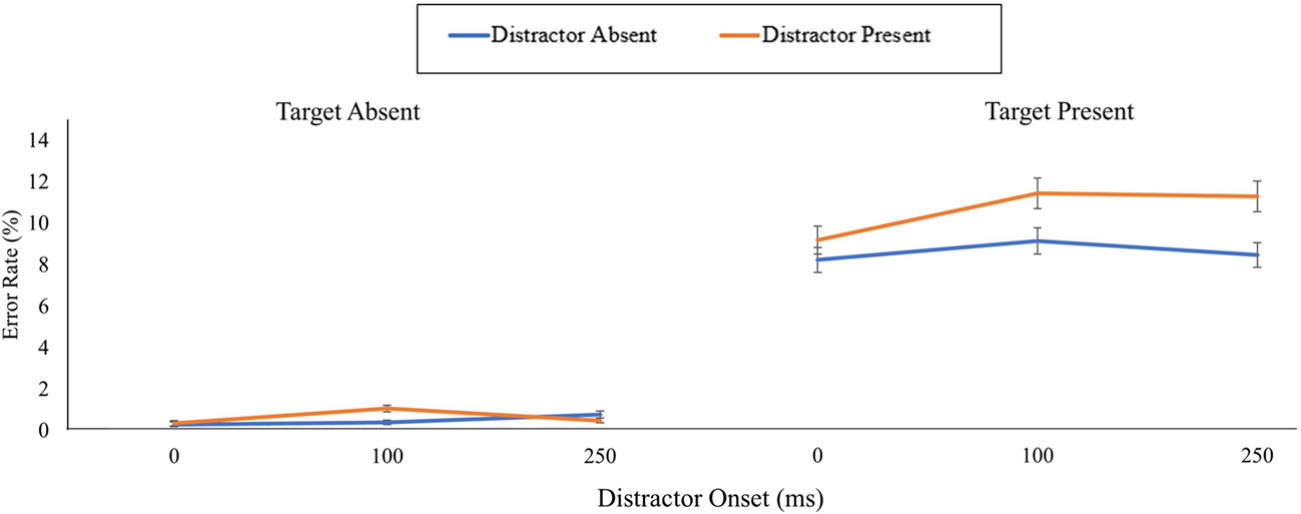
The mean error rates across each combination of target, distractor, and distractor onset of Experiment 1. The error bars represent the standard error of the mean corrected to remove between-subject variability (Cousineau, 2005). (Colour figure online)

First, the error rate data for target-present trials were entered into a 2 (distractor [present, absent]) by 3 (distractor onset block [0 ms, 100 ms, 250 ms]) repeated-measures ANOVA (Fig. 2). Overall, there was a main effect of distractor presence, *F*(1, 83) = 17.19, *p* < .001, η_p_^2^ = .17 (BF_10_ = 29.94), where error rates were higher when the distractor was present (*M* = 10.54%) compared with absent (*M* = 8.52%). However, there was no main effect of distractor onset block, *F*(2, 166) = 1.90, *p* = .153, η_p_^2^ = .02 (BF_10_ = 0.22), nor was there a significant interaction between distractor and distractor onset block, *F*(2, 166) = 1.08, *p* = .341, η_p_^2^ = .01 (BF_10_ = 0.13).

**Fig. 3:**
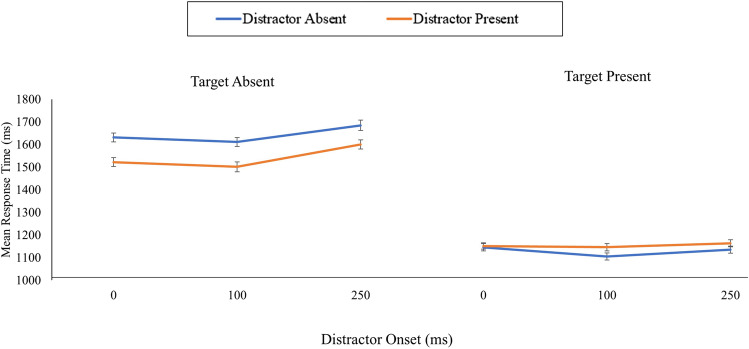
The mean response times across each combination of target, distractor, and distractor onset of Experiment 1. The error bars represent the standard error of the mean corrected to remove between-subject variability (Cousineau, 2005). (Colour figure online)

Second, the RT data for correct trials were entered into a 2 (distractor) by 3 (distractor onset block) repeated-measures ANOVA (Fig. 3). Again, there was a main effect of distractor presence on correct RTs, *F*(1, 83) = 10.19, *p* = .002, η_p_^2^ = .11 (BF_10_ = 2.05), where RTs were slower for distractor-present trials (*M* = 1,154 ms) compared with distractor-absent trials (*M* = 1129 ms). Similarly, there was no main effect of distractor onset block, *F*(2, 166) = 0.69, *p* = .505, η_p_^2^ = .01 (BF_10_ = 0.14), nor was there an interaction between distractor presence and distractor onset block, *F*(2, 166) = 1.19, *p* = .305, η_p_^2^ = .01 (BF_10_ = 0.16).

#### Target-absent trials

Having explored the data for target-present trials, we next turned our attention to the main condition of interest, target-absent trials. First, error rates for target-absent trials were entered into a 2 (distractor) by 3 (distractor onset block) repeated-measures ANOVA (Fig. 2). Although the main effect of distractor was non-significant, *F*(1, 83) = 1.60, *p* = .210, η_p_^2^ = .02 (BF_10_ = 0.21), the main effect of distractor onset block was significant, *F*(2, 166) = 3.59, *p* = .030, η_p_^2^ = .04 (BF_10_ = 0.75), as was the interaction between distractor and distractor onset block, *F*(2, 166) = 6.07, *p* = .003, η_p_^2^ = .07 (BF_10_ = 24.13). Follow-up paired-samples *t* tests revealed a significant effect of distractor presence for the 100ms onset condition, *t*(83) = 3.31, *p* = .001, *d* = 0.36 (BF_10_ = 17.78), where error rates were higher for distractor-present (*M* = 1.05%) compared with distractor-absent trials (*M* = 0.38%). However, there was no significant difference for the 0ms condition, *t*(83) = 0.28, *p* = .783, *d* = 0.03, (BF_10_ = 0.13), nor for the 250-ms condition, *t*(83) = 1.35, *p* = .181, *d* = 0.15 (BF_10_ = 0.29).

For the correct RT data (Figure 3), there was a main effect of distractor, *F*(1, 83) = 68.98, *p* < .001, η_p_^2^ = .45 (BF_10_ = 3.06 × 10^9^), where RTs were faster for distractor-present trials (*M* = 1,541 ms) compared with distractor-absent trials (*M* =1,642 ms). Furthermore, there was also a main effect of distractor onset block, *F*(2, 166) = 3.92, *p* = .022, η_p_^2^ = .05 (BF_10_ = 2.47; *M *_*0 ms*_* =* 1,577 ms, *M *_*100 ms*_* =* 1,556 ms, *M *_*250 ms*_* =* 1,642 ms). However, there was no interaction between distractor and distractor onset block, *F*(2, 166) = 0.78, *p* = .462, η_p_^2^ = .01 (BF_10_ = 0.09).

### Discussion

Experiment 1 tested if the QTE was modulated by the timing of the distractor onset. Overall, the distractor onset block did not appear to alter the magnitude of the QTE. That is, the presence of the distractor increased error rates for target-present visual search and speeded target-absent visual search, with onset having minimal influence on that effect. Although there was an interaction between distractor and distractor onset block for error rates on target-absent trials, given that error rates were extremely low overall, and that error rates on target-absent trials are not paramount to the QTE, it seems likely that a delayed onset is not a necessary condition for lowered quitting thresholds under distraction.

## Experiment 2

In Experiment 2, we evaluated whether the findings of Experiment 1 were observable upon replication, as well as introduced a condition where the salient distractor appeared 100 ms before all other items in the search array. Indeed, prior work has demonstrated that when some of the distractor items onset prior to the target and the rest of the distractors, search is performed faster than when all the distractors onset with the target, which has been termed the visual marking effect (Watson & Humphreys, 2000, 2002; Watson et al., 2003). If participants were first exposed to the distractor before other items, they may be able to suppress the distractor, thus further lowering its salience. This may reduce the QTE compared with a simultaneous and delayed onset condition.

### Method

#### Participants, materials, and procedure

One hundred and one participants completed a demographic survey and the online visual search task. One participant did not provide any demographic information. For the remaining participants, the mean age was 21.44 years (*SD* = 5.32 years). Eighty-seven were female, 12 were male, and one reported other. Eighty-nine were right-handed, seven were left-handed and four were ambidextrous. All reported normal or corrected-to-normal vision, provided informed consent, and were compensated with course credit for an undergraduate psychology course. The materials and procedures used in Experiment 2 were similar to Experiment 1 except for the following. First, instead of onsets of 0 ms, 100 ms, and 250 ms, onsets of −100 ms, 0 ms, and 100 ms were adopted. Second, instead of onset being blocked, distractor onset was randomly intermixed across one block of 12 practice trials and 360 experimental trials which were broken into three 120-trial blocks. Please note that for statistical analyses, this meant that the effect of experimental block could not be used to test the effect of distractor onset. Therefore, for each participant, each distractor absent trial was matched to one of the three distractor onset conditions, so that there would be an equal number of “distractor present” compared with “distractor absent” trials to analyse for each onset condition.

### Results

#### Data cleaning

The same data cleaning methods were used in Experiment 2 as in Experiment 1. First, participants were removed if they achieved less than 60% accuracy overall, or if they achieved less than 10% accuracy in any one experimental condition (*N* = 2; Moher, 2020). Next, any participants with extreme scores in error rates for any condition relative to the sample (i.e., *SD =* ±3.29) were removed (*N* = 8). For the remaining 91 participants, we next looked at correct RTs. At the individual level, first, we removed observations for each participant that were faster than 200ms or slower than 10 seconds. On average, this removed 0.31% (*SD* = 0.97%) of correct trials per participant. Finally, we checked for univariate outliers on mean RT for each condition (±3.29 *SD*s). Four outliers were identified and removed from further analyses, leaving a final sample of 87 participants.

#### Target-present trials

**Fig. 4:**
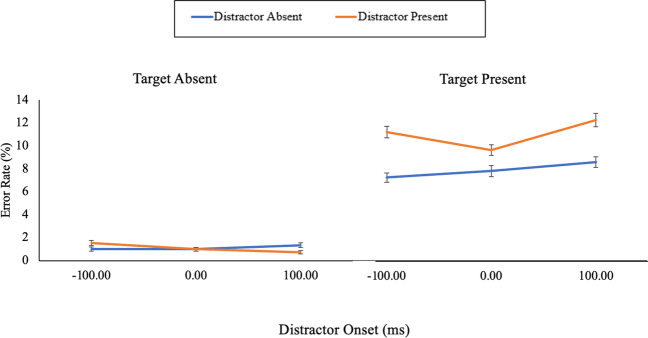
The mean error percentages across each combination of target, distractor, and distractor onset of Experiment 2. The error bars represent the standard error of the mean corrected to remove between-subject variability (Cousineau, 2005). (Colour figure online)

The error rate and correct RT data for Experiment 2 were analysed in the same way as in Experiment 1 by using 2 (distractor) by 3 (distractor onset) repeated-measures ANOVAs. Firstly, for error rates (Fig. 4), there was a main effect of distractor presence, *F*(1, 86) = 43.75, *p* < .001, η_p_^2^ = .34 (BF_10_ = 1.44 × 10^6^; *M*_present_ = 11.88%, *M*_absent_ = 8.51%), a main effect of distractor onset, *F*(2, 172) = 4.79, *p* = .009, η_p_^2^ = .05, (BF_10_ = 1.89; *M *_*-100 ms*_* =* 9.94%, *M *_*0 ms*_* =* 9.41%, *M *_*100 ms*_* =* 11.23%), and a non-significant interaction between distractor presence and distractor onset, *F*(2, 172) = 2.72, *p* = .069, η_p_^2^ = .03 (BF_10_ = 0.62).

**Fig. 5:**
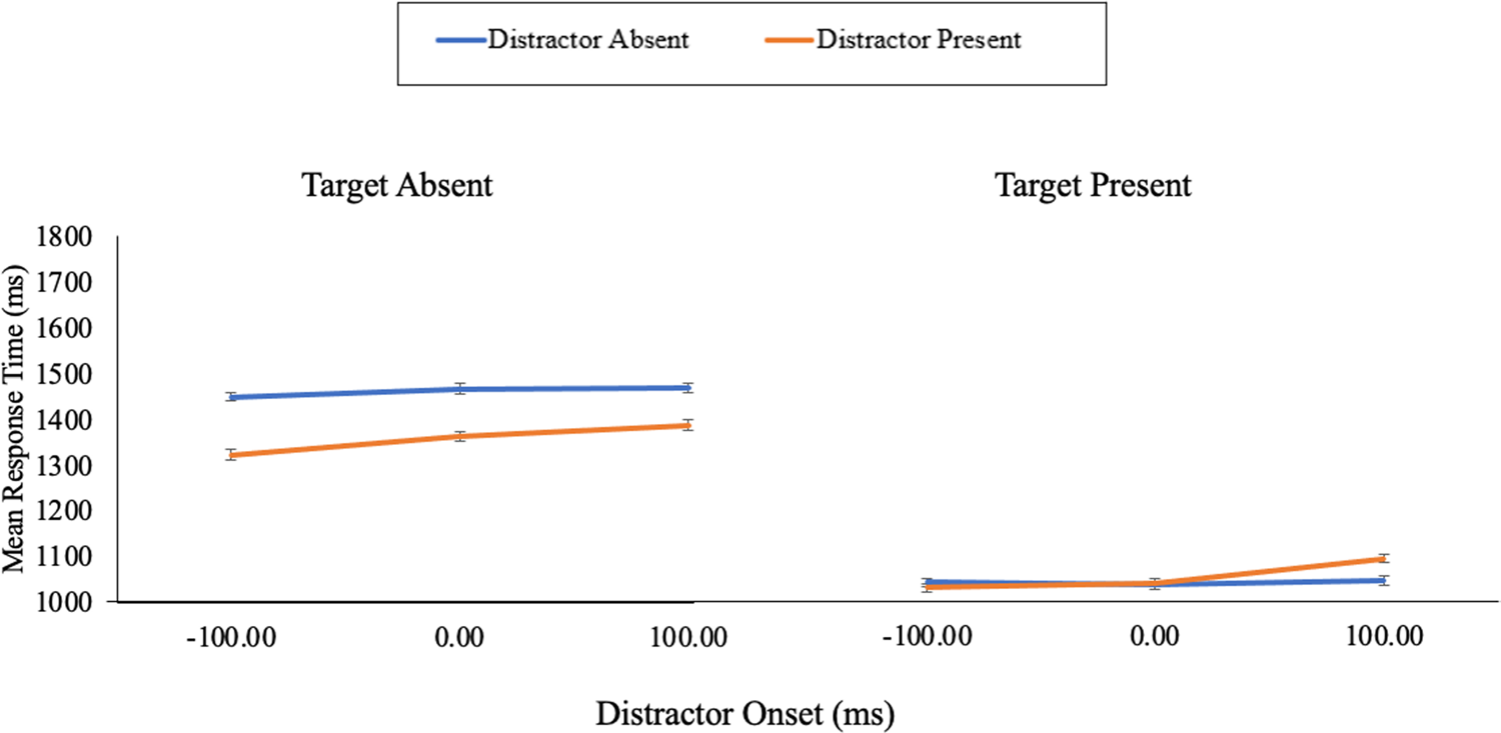
The mean response times across each combination of target, distractor, and distractor onset of Experiment 2. The error bars represent the standard error of the mean corrected to remove between-subject variability (Cousineau, 2005). (Colour figure online)

Secondly, for the correct RT data (Fig. 5), the main effect of distractor presence was nonsignificant, *F*(1, 86) = 2.70, *p* = .104, η_p_^2^ = .03 (BF_10_ = 0.37), and the main effect of distractor onset was significant, *F*(2, 172) = 10.39, *p* < .001, η_p_^2^ = .11 (BF_10_ = 19.98). Critically, the interaction between distractor presence and distractor onset was significant, *F*(2, 172) = 4.30, *p* = .015, η_p_^2^ = .05 (BF_10_ = 7.35). For the −100-ms onset condition, there was no significant difference in RTs for the distractor-present (*M* = 1,031 ms) and distractor-absent (*M* = 1,042 ms) trials, *t*(86) = 0.85, *p* = .395, *d* = 0.09 (BF_10_ = 0.17). There was also no significant difference in RTs for distractor-present (*M* = 1042 ms) and distractor-absent (*M* = 1036 ms) trials for the 0-ms onset condition, *t*(86) = 0.40, *p* = .690, *d* = 0.04 (BF_10_ = 0.13). However, there was a significant difference in RTs for distractor-present (*M* = 1,095 ms) and distractor-absent (*M* = 1,047 ms) trials for the 100-ms onset condition, *t*(86) = 2.82, *p* = .006, *d* = 0.30 (BF_10_ = 4.70).

#### Target-absent trials

For the error rate data, there was no main effect of distractor presence, *F*(1, 86) = 0.02, *p* = .883, η_p_^2^ < .01 (BF_10_ = 0.12), and no main effect of distractor onset,* F*(1.84, 158.35) = 1.15, *p* = .317, η_p_^2^ = .01 (BF_10_ = 0.07). However, there was a significant interaction between distractor presence and distractor onset, *F*(2, 172) = 4.12, *p* = .018, η_p_^2^ = .05 (BF_10_ = 3.76). Paired-samples *t* tests revealed that for the −100 ms condition, there was no significant difference in error rates for distractor-present (*M =* 1.53%) compared with distractor-absent (*M* = 1.00%) trials, *t*(86) =1.64, *p* = .104, *d* = 0.18 (BF_10_ = 0.43). In the 0-ms condition, there was also no significant difference in error rates for distractor-present (*M =* 1.00%) and distractor-absent (*M =* 1.00%) trials, *t*(86) < 0.01, *p* > .999, *d* < 0.01 (BF_10_ = 0.12). However, for the 100-ms condition, there was a significant difference in error rates for distractor-present (*M =* 0.73%) and distractor-absent (*M =* 1.34%) trials, *t*(86) =2.14, *p* = .035, *d* = 0.23 (BF_10_ = 1.04).

For the correct RT data, there was a main effect of distractor, *F*(1, 86) = 72.84, *p* < .001, η_p_^2^ = .46 (BF_10_ = 1.66 × 10^10^), where RTs were faster for distractor-present (*M* = 1,358 ms) compared with distractor-absent (*M* = 1,462 ms) trials. There was also a main effect of distractor onset, *F*(2, 172) = 6.90, *p* = .001, η_p_^2^ = .07 (BF_10_ = 9.85; *M *_*-100 ms*_* =* 1,387 ms *M *_*0 ms*_* =* 1,414 ms, *M *_*100 ms*_* =* 1,429 ms). Nonetheless, the interaction between distractor presence and distractor onset was nonsignificant, *F*(1.86, 160.01) = 2.41, *p* = .097, η_p_^2^ = .03 (BF_10_ = 0.41).

### Discussion

Experiment 2 tested if an early onset distractor produces a similar QTE to that seen with simultaneous and delayed onset distractors during visual search. Distractor onsets of -100 ms, 0 ms and 100 ms were used. Overall, the QTE emerged for all three distractor onsets. That is, regardless of onset, the presence of the distractor increased error rates for target-present trials and speeded RTs for target-absent trials. Nonetheless, it is important to note that the distractor had differing effects on both target-absent error rates and target-present reaction times across the three onset conditions. Specifically, the delayed onset distractor seemed to have a greater effect on performance in these conditions compared with the early and simultaneous onset. Taken together, this suggests that while changes in onset can (and do) seem to impact distractor salience, changes in salience via a delayed onset do not appear to be necessary for generating the distractor QTE.

## General discussion

The current study explored whether the timing of a salient distractor modulates the QTE originally observed by Moher during target detection visual search. In Experiment 1, the onset of the distractor was either simultaneous with other array items or delayed by 100 ms or 250 ms. In Experiment 2, the distractor onset was either 100 ms prior to the main array, simultaneous with the other array items, or delayed by 100 ms. Across both experiments, clear QTEs were observed: regardless of onset, the distractor increased errors on target-present trials and decreased reaction times on target-absent trials. As such, it appears that a salient singleton distractor does not need to have a delayed onset for a distractor QTE to emerge.

Previous work by Lawrence and Pratt (2022) suggests that distractor salience modulates the QTE. In their study, salience was manipulated using size. Specifically, the QTE only emerged when a large, delayed-onset colour distractor was used compared with a small, delayed-onset colour distractor. Here, we found a QTE when a large colour distractor was deployed, regardless of the time it appeared in the search array. Given that changes in onset are thought to modulate salience, and that salience modulates the QTE, this finding might seem surprising at face value. Nonetheless, it is worth noting that in Experiment 2 of the current study, there was a significant interaction between distractor presence and onset for target-present trials. Specifically, distractor presence only appeared to slow RTs for the 100-ms delayed onset condition (not the −100-ms and 0-ms onset conditions). This finding suggests that the onset manipulation was indeed influencing distractor salience in this study (at least in Experiment 2), but that this change in salience was not necessary for seeing the QTE in target-absent RTs and target-present error rates. That is, perhaps the size and colour manipulations of salience are so strong that any small changes in salience generated by differing onsets result in only minimal effects on the QTE, while still impacting performance on target-present trials.

On a related note, one might have expected that when a distractor was shown 100 ms prior to the onset of other array items, participants may have been better able to suppress the processing of the salient distractor, lower reinspection rates, and, in turn, minimise the QTE. However, this was not observed. Even when given advance notice of the distractor (−100 ms onset block in Experiment 2), a QTE emerged. This finding supports the possibility that, regardless of onset, distractors lower quitting thresholds potentially due to being inspected, rejected, and then reinspected multiple times during search (e.g., Horowitz & Wolfe, 1998; Lawrence & Pratt, 2022; Moher, 2020; Moran et al., 2013; Wolfe, 2021).

Nonetheless, for the 0ms and 100ms onset conditions, RTs varied across experiments, with participants responding faster in Experiment 2 compared with Experiment 1. One reason this might have occurred is due to the different designs used in Experiments 1 and 2. Specifically, in Experiment 1, distractor onset was blocked and the distractor appeared either at the same time as other array items or at a delayed time. In contrast, distractor onset was intermixed within a block for Experiment 2, with one of the onsets being *before* other items in the array appeared. Critically, these two designs may have helped participants establish different strategies (or expectations) for distractor processing. For example, in Experiment 2 participants could not anticipate the timing of the distractor, and as such may have been expecting (and then preparing) to suppress the distractor prior to the onset of the other items in the array, resulting in faster RTs overall. 

Finally, it is worth considering the results of the current study in a more applied sense. Target detection visual search tasks are commonly undertaken in military, security, and police operations. As such, there is a clear need to apply findings from basic cognitive psychological research to these settings (Biggs et al., 2018). Here, we have shown that in a lab-based search task, delayed distractor onsets are not crucial for generating the QTE. This is an important finding practically, as it would not always be the case in an applied setting for a distractor to suddenly appear and capture attention. Instead, it appears that if a distractor is sufficiently salient, QTEs can emerge.

## Footnotes

1 As is typical of online studies, there was some data loss. In both Experiments, a greater number of participants completed the demographic survey than completed both the demographic survey and computer task. Furthermore, the demographic survey and computer task were hosted on two separate websites (Lime Survey and Pavlovia). Therefore, at the end of the demographic survey, participants were provided with a random 5-digit number to enter on Pavlovia that was matched to the data. In the current experiments, we only report data from participants whose data could be matched using this method.
